# Complete Genome Sequence of *Lactobacillus acidophilus* MN-BM-F01

**DOI:** 10.1128/genomeA.01699-15

**Published:** 2016-02-11

**Authors:** Lan Yang, Yun Chen, Zhiwei Li, Yudong Shi, Zhouyong Li, Xiaohui Zhao

**Affiliations:** Research and Development Center, Inner Mongolia Mengniu Dairy (Group) Co., Ltd., Hohhot, Inner Mongolia, China

## Abstract

*Lactobacillus acidophilus* MN-BM-F01 was originally isolated from a traditional fermented dairy product in China. The characteristics of this bacterium are its low post-acidification ability and high acid-producing rate. Here, we report the main genome features of *L. acidophilus* MN-BM-F01.

## GENOME ANNOUNCEMENT

*Lactobacillus acidophilus* is a lactic acid bacterium widely used by the dairy industry as a starter culture to obtain high-quality fermented products (1–3), such as cheeses and yogurt. *L. acidophilus* MN-BM-F01 was isolated from a traditional fermented dairy product called dairy fan, in the region of Yunnan province, China. The low post-acidification ability and high acid-producing rate of *L. acidophilus* are useful for dairy products, which can extend shelf life and shorten the milk coagulation time.

Whole-genome sequencing of *L. acidophilus* MN-BM-F01 was performed using the whole-genome shotgun strategy with Pacific Biosciences RS II sequencing platform (Pacific Biosciences, Menlo Park, CA, USA). A 10-kb single-molecule real-time (SMRT) bell library was prepared from sheared genomic DNA using a 10-kb template library preparation workflow. SMRT sequencing was conducted on a PacBio RS II sequencing platform using the C4 sequencing chemistry and P6 polymerase with 1 SMRT cell. *De novo* assembly of the PacBio read sequences was carried out using continuous long reads following the Hierarchical Genome Assembly Process (HGAP) workflow (PacBioDevNet; Pacific Biosciences) as available in SMRT Analysis version 2.1. The complete genome sequence of *L. acidophilus* MN-BM-F01 contains a circular 1,875,071-bp chromosome, with a GC content of 49.71%. In total, there are 2,121 predicted genes in the chromosome, including 2,010 protein-encoding genes, 87 tRNA-encoding genes, and 24 rRNA-encoding genes (4, 5).

The functions of predicted proteins were annotated based on the comparison of homologues to the NCBI-nr, Pfam, and KEGG databases. We have found that, of all the proteins in *L. acidophilus* MN-BM-F01, 507 have homologues in the eggNOG databases and have been assigned proper terms. The remaining have no orthologous groups (with E-value <1E−5). Gene Ontology and KEGG analyses were performed by Blast2GO (6), and 981 and 968 genes, respectively, were assigned to proper terms.

### Nucleotide sequence accession number.

The sequence and annotation of the *Lactobacillus acidophilus* MN-BM-F01 genome is available from GenBank under accession number CP013610.
