# Correction for Yang et al., Complete Genome Sequence of *Lactobacillus delbrueckii* subsp. *bulgaricus* MN-BM-F01

**DOI:** 10.1128/genomeA.00420-16

**Published:** 2016-05-12

**Authors:** Lan Yang, Yun Chen, Zhiwei Li, Yudong Shi, Zhouyong Li, Xiaohui Zhao

**Affiliations:** Research and Development Center, Inner Mongolia Mengniu Dairy (Group) Co., Ltd., Hohhot, Inner Mongolia, China

## AUTHOR CORRECTION

Volume 4, no. 1, e01699-15, 2016. Page 1: The title should read as given above, and strain MN-BM-F01 should be identified throughout as *Lactobacillus delbrueckii* subsp. *bulgaricus* rather than *Lactobacillus acidophilus.*

